# XRCC3 Thr241Met gene polymorphisms and lung cancer risk: a meta-analysis

**DOI:** 10.1186/1756-9966-32-1

**Published:** 2013-01-04

**Authors:** Ping Zhan, Qin Wang, Qian Qian, Li-Ke Yu

**Affiliations:** 1 First Department of Respiratory Medicine, Nanjing Chest Hospital, 215 Guangzhou Road, Nanjing, 210029, China; 2 Department of Respiratory Medicine, 81 Hospital of PLA, Nanjing, China

**Keywords:** XRCC3, Polymorphism, Lung cancer, Susceptibility, Meta-analysis

## Abstract

Many studies have examined the association between the XRCC3 Thr241Met gene polymorphism and lung cancer risk in various populations, but their results have been inconsistent. To assess this relationship more precisely, a meta-analysis was performed. The PubMed, Embase, Web of Science, and CNKI database was searched for case–control studies published up to July 2012. Data were extracted and pooled odds ratios (OR) with 95% confidence intervals (CI) were calculated.

Ultimately, 17 studies, comprising 4123 lung cancer cases and 5597 controls were included. Overall, for T allele carriers (TC + TT) versus the wild-type homozygotes (CC), the pooled OR was 0.95 (95% CI = 0.87-1.04 *P* = 0.228 for heterogeneity), for TT versus CC the pooled OR was 0.99 (95% CI = 0.86-1.15 *P* = 0.315 for heterogeneity). In the stratified analysis by ethnicity, histological types of lung cancer and smoking status, no any significantly risks were found for (C/T + T/T) vs C/C or T/T vs C/C. No publication bias was found by using the funnel plot and Egger's test.

Overall, there is no evidence showing a significant correlation between XRCC3 Thr241Met polymorphism and lung cancer risk stratified analysis by ethnicity, histology and smoking status.

## Introduction

Lung cancer remains the most lethal cancer worldwide, despite improvements in diagnostic and therapeutic techniques [1]. Its incidence has not peaked in many parts of world, particularly in China, which has become a major public health challenge all the world [2]. The mechanism of lung carcinogenesis is not understood. Although smoking status is the single most important factor that causes lung cancer, host factors including genetic polymorphism, had garnered interest with regard to the study of the tumorigenesis of lung cancer [3]. Otherwise, accumulating studies have suggested that lung cancers occurring in never smokers have different molecular profiles. In this way, host genetic susceptibility is a very important factor in the development of lung cancer, contributing to the variation in individual cancer risk. DNA repair gene system plays a crucial role in protecting against gene mutation caused by tobacco smoke. Recent studies have revealed that single nucleotide polymorphisms (SNPs) in DNA repair genes may be the underlying molecular mechanism of the individual variation of DNA repair capacity [4,5]. Increasing molecular epidemiologic evidence has shown that polymorphisms in various DNA repair genes are associated with an increased risk of lung cancer [6,7].

The X-ray repair cross-complementing group 3 (XRCC3) belongs to a family of genes responsible for repairing DNA double strand breaks caused by normal metabolic processes and/or exposure to ionizing radiation [8].The XRCC3 gene codes for a protein involved in homologous recombinational repair (HRR) for double strand breaks of DNA (DBSs) and cross-link repair in mammalian cells [9]. During HRR, the XRCC3 protein interacts with Rad51 protein and likely contributes to maintain chromosome stability. A common polymorphism in exon 7 of the XRCC3 gene results in an amino acid substitution at codon 241 (Thr241Met) that may affect the enzyme function and/or its interaction with other proteins involved in DNA damage and repair [10]. The predominant homozygous allele, the heterozygous allele and the homozygous rare allele of the XRCC3 Thr241Met gene polymorphism are named the homozygous wild-type genotype (C/C), the heterozygote (C/T) and the homozygote (T/T), respectively.

Recently, many studies have investigated the role of the XRCC3 Thr241Met gene polymorphism in lung cancer. However, the results of these studies remain inconclusive. A single study might not be powered sufficiently to detect a small effect of the polymorphisms on lung cancer, particularly in relatively small sample sizes. Further, past studies have not controlled for the potential confounding effect of smoking properly-the main risk determinant for lung cancer. Various types of study populations and study designs might also have contributed to these disparate findings. To clarify the effect of the XRCC3 Thr241Met gene polymorphism on the risk for lung cancer, we performed a meta-analysis of all eligible case–control studies that have been published and conducted the subgroup analysis by stratification according to the ethnicity source, histological types of lung caner and smoking status of case and control population.

## Materials and methods

### Publication search

We searched for studies in the PubMed, Embase, Web of Science, and CNKI (China National Knowledge Infrastructure) electronic databases to include in this meta-analysis, using the terms “XRCC3,” “X-ray repair cross-complementing group 3,” “polymorphism,” and “lung cancer.” An upper date limit of July 01, 2012 was applied; no lower date limit was used. The search was performed without any restrictions on language and was focused on studies that had been conducted in humans. We also reviewed the Cochrane Library for relevant articles. Concurrently, the reference lists of reviews and retrieved articles were searched manually. Only full-text articles were included. When the same patient population appeared in several publications, only the most recent or complete study was included in this meta-analysis.

### Inclusion criteria

For inclusion, the studies must have met the following criteria: they (1) evaluated XRCC3 gene polymorphisms and lung cancer risk; (2) were case–control studies; (3) supplied the number of individual genotypes for the XRCC3 Thr241Met gene polymorphisms in lung cancer cases and controls, respectively; and (4) demonstrated that the distribution of genotypes among controls were in Hardy-Weinberg equilibrium.

### Data extraction

Information was extracted carefully from all eligible publications independently by 2 authors, based on the inclusion criteria above. Disagreements were resolved through a discussion between the 2 authors.

The following data were collected from each study: first author’s surname, year of publication, ethnicity, total numbers of cases and controls, and numbers of cases and controls who harbored the XRCC3 Thr241Met genotypes, respectively. We did not contact the author of the primary study to request the information. Ethnicities were categorized as Asian, Caucasian, and mixed population. Histological type of lung cancer was divided to lung squamous carcinoma (SCC), adenocarcinoma (AC) and small cell lung cancer (SCLC) in our meta-analysis. The definition of smoking history is very complicated. The smoking histories covered different periods if changes in the number of cigarettes smoked per day or type of tobacco products occurred. According to the general standards, non-smokers were defined as subjects who had smoked less than 100 cigarettes in their lifetime. Although the precise definition of never-smoking status varied slightly among the studies, the smoking status was classified as non-smokers (or never smoker) and smokers (regardless of the extent of smoking) in our meta-analysis. We did not require a minimum number of patients for a study to be included in our meta-analysis.

### Statistical analysis

OR (odds ratios) with 95% CIs were used to determine the strength of association between the XRCC3 Thr241Met polymorphisms and lung cancer risk.

The pooled ORs for the risk associated with the XRCC3 Thr241Met genotype, the T allele carriers (TC + TT) versus the wild-type homozygotes (CC), TT versus CC were calculated, respectively. Subgroup analyses were done by ethnicity, histological types of lung cancer and smoking status. Heterogeneity assumptions were assessed by chi-square-based Q-test [11]. A *P* value greater than 0.10 for the Q-test indicated a lack of heterogeneity among the studies. Thus, the pooled OR estimate of each study was calculated using the fixed-effects model (the Mantel-Haenszel method) [12]; otherwise, the random-effects model (the DerSimonian and Laird method) was used [13].

One-way sensitivity analyses were performed to determine the stability of the results-each individual study in the meta-analysis was omitted to reflect the influence of the individual dataset on the pooled OR [14].

Potential publication biases were estimated by funnel plot, in which the standard error of log (OR) of each study was plotted against its log (OR). An asymmetrical plot suggests a publication bias. Funnel plot asymmetry was assessed by Egger’s linear regression test, a linear regression approach that measures the funnel plot asymmetry on a natural logarithm scale of the OR. The significance of the intercept was determined by t-test, as suggested by Egger (P < 0.05 was considered a statistically significant publication bias) [15].

All calculations were performed using STATA, version 11.0 (Stata Corporation, College Station, TX).

## Results

### Study characteristics

A total of seventeen publications involving 4123 lung cancer cases and 5597 controls met the inclusion criteria and were ultimately analyzed [16-31]. Table 1 presents the main characteristics of these studies. David-Beabes 's study [16] sorted the data for Caucasians and Mixed populations; therefore, each group in the study was considered separately in the pooled subgroup analyses.

**Table 1:** Distribution of XRCC3 Thr241Met genotypes among lung cancer cases and controls included in this meta-analysis

**First author-year**	**Ethnicity(country of origin)**	**Total sample size (case/control)**	**Lung cancer cases**			**Controls**		
			**C/C**	**C/T**	**T/T**	**C/C**	**C/T**	**T/T**
David-Beabes-2001	USA (Caucasian)	178/453	76	78	24	175	210	68
David-Beabes-2001	USA (Mixed)	153/234	90	54	9	136	88	10
Misra-2001	Finland (Caucasian)	313/306	160	124	29	149	134	23
Wang-2003	USA (Mixed)	112/190	69	43^#^		119	71^#^	
Popanda-2004	Germany (Caucasian)	462/459	175	201	86	168	222	69
Jacobsen-2004	Denmark (Caucasian)	246/269	95	123	28	113	113	43
Harms-2004	USA (Caucasian)	110/119	61	37	12	61	49	9
Matullo-2006	Europe (Caucasian)	116/1094	44	56	16	383	544	167
Zienolddiny-2006	Norway (Caucasian)	220/250	114	90	16	115	111	24
Rky-2006	Sweden (Caucasian)	175/154	79	96^#^		56	98^#^	
Lopez-Cima-2007	Spain (Caucasian)	403/434	168	185	50	178	196	60
Zhang-2007	China (Asian)	291/273	259	30	2	244	28	1
Improta-2008	Italy (Caucasian)	94/121	31	33	30	67	46	8
Xia-2008	China (Asian)	103/139	91	12	0	118	21	0
Osawa K-2010	Japan (Asian)	104/120	92	12^#^		98	22^#^	
Qian B-2011	China (Asian)	581/603	521	60	0	533	67	3
Kiyohara C-2012	Japan (Asian)	462/379	352	97	13	295	77	7

^#^, the number of the combined C/T and T/T genotypes.

Of the 17 publications, 15 were published in English and only 2 were written in Chinese. The sample sizes ranged from 185 to 1210. All cases were histologically confirmed. The controls were primarily healthy populations and matched for age, ethnicity, and smoking status. There were 5 groups of Asians, 10 groups of Caucasians, and 2 mixed populations. All polymorphisms in the control subjects were in Hardy-Weinberg equilibrium.

### Meta-analysis results

**Figure 1:**
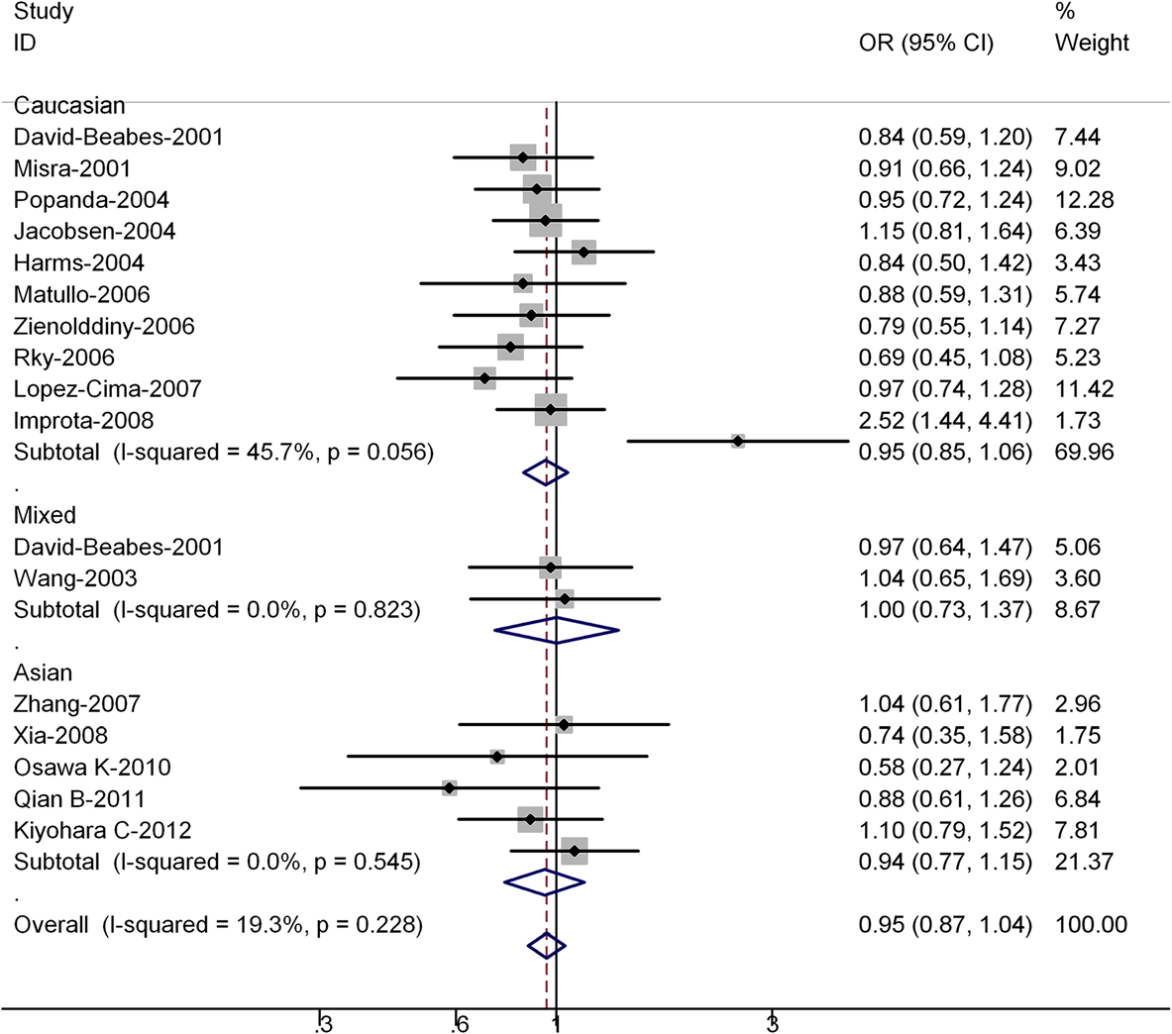
**Forest plot (random-effects model) of lung cancer risk associated with XRCC3 Thr241Met polymorphisms for the (C/T + T/T) versus vs C/C.** Each box represents the OR point estimate, and its area is proportional to the weight of the study. The diamond (and broken line) represents the overall summary estimate, with CI represented by its width. The unbroken vertical line is set at the null value (OR =1.0).

Table 2 listed the main results of this meta-analysis. Overall, for the T allele carriers (TC + TT) versus homozygote CC, the pooled OR for all studies combined 4123 cases and 5597 controls was 0.95 (95% CI = 0.87-1.04 *P* = 0.228 for heterogeneity) (Figure 1), for TT versus CC the pooled OR was 0.99 (95% CI = 0.86-1.15 *P* = 0.315 for heterogeneity). For all studies in the meta-analysis, significantly risks were not found for the T allele carriers (TC + TT) versus homozygote CC or TT versus CC, and no heterogeneity was found in all studies.

**Table 2:** Summary ORs for various contrasts of XRCC3 Thr241Met gene polymorphisms in this meta-analysis

**Subgroup analysis**	**exon7 genotype**		
	**Contrast studies OR(95%) P**_**h**_		
**Total**	T/T vs C/C	17	0.99(0.86-1.15) 0.315
	(C/T + T/T) vs C/C		0.95(0.87-1.04) 0.228
Ethnicity			
Asian	T/T vs C/C	5	0.92(0.71-1.09) 0.216
	(C/T + T/T) vs C/C		0.94(0.77-1.15) 0.545
Caucasian	T/T vs C/C	10	0.94(0.87-1.13) 0.090
	(C/T + T/T) vs C/C		0.95(0.85-1.06) 0.056
Mixed population	T/T vs C/C	2	1.04(0.77-1.43) 0.190
	(C/T + T/T) vs C/C		1.00(0.73-1.37) 0.823
**Histological type**			
SCC	T/T vs C/C	2	0.94(0.78-1.58) 0.164
	(C/T + T/T) vs C/C		0.91(0.48-1.74) 0.215
AC	T/T vs C/C	3	1.09(0.72-1.38) 0.535
	(C/T + T/T) vs C/C		1.05(0.79-1.40) 0.331
**Smoking status**			
Smoker	T/T vs C/C	4	0.98(0.72-1.45) 0.006
	(C/T + T/T) vs C/C		0.93(0.63-1.37) 0.001
Non-smoker	T/T vs C/C	4	0.99(0.78-1.51) 0.230
	(C/T + T/T) vs C/C		0.92(0.62-1.37) 0.186

P_h_ P value of Q-test for heterogeneity test.

In the stratified analysis by ethnicity, significantly risks were not found among Asians for (TC + TT) versus CC (OR = 0.94, 95% CI = 0.77-1.15; *P* = 0.545 for heterogeneity) or TT versus CC (OR = 0.92; 95% CI = 0.71-1.09; *P* = 0.216 for heterogeneity). For Caucasians, significantly risks were not found for (TC + TT) versus CC (OR = 0.95, 95% CI = 0.85-1.06; *P* = 0.056 for heterogeneity) or TT versus CC (OR = 0.94; 95% CI = 0.87-1.13; *P* = 0.090 for heterogeneity) .

**Figure 2:**
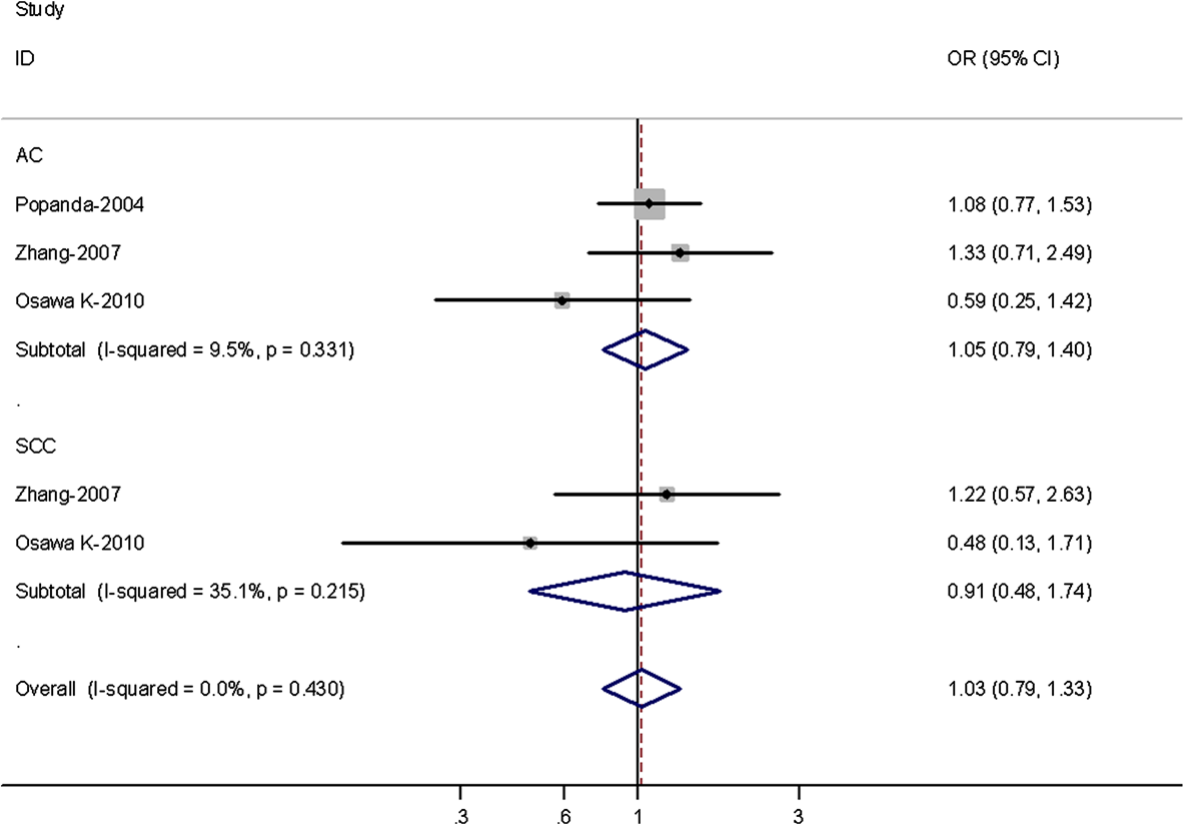
Forest plot (random-effects model) of lung cancer risk associated with XRCC3 Thr241Met polymorphisms for the (C/T + T/T) versus vs C/C stratified by histological types of lung cancer.

Three out of 17 studies examined the association of XRCC3 Thr241Met genotype and the risk of different histological types of lung cancer including SCC and AC (Table 3). Among lung SCC, no significantly increased risks were observed for (TC + TT) versus CC (OR = 0.91, 95% CI = 0.48-1.74; *P* = 0.215 for heterogeneity) or TT versus CC (OR = 0.94; 95% CI = 0.78-1.58; *P* = 0.164 for heterogeneity). Among lung AC, no significant associations were observed for both (TC + TT) versus CC or TT versus CC (Figure 2).

**Table 3:** Distribution of XRCC3 Thr241Met genotypes among cases and controls stratified by histological types of lung cancer

**First author-year**	**Ethnicity(country of origin)**	**Histology (Scc/Ac/Sclc)**	**Lung cancer cases**			**Controls**		
			**C/C**	**C/T**	**T/T**	**C/C**	**C/T**	**T/T**
Popanda-2004	Germany (Caucasian)	AC	71	89	44	168	222	69
Zhang-2007	China (Asian)	AC	114	18^#^		244	29^#^	
		SCC	69	10^#^		244	29^#^	
Osawa K-2010	Japan (Asian)	AC	60	8^#^		98^#^	22^#^	
		SCC	28	3^#^		98^#^	22^#^	

^#^, the number of the combined C/T and T/T genotypes.

**Figure 3:**
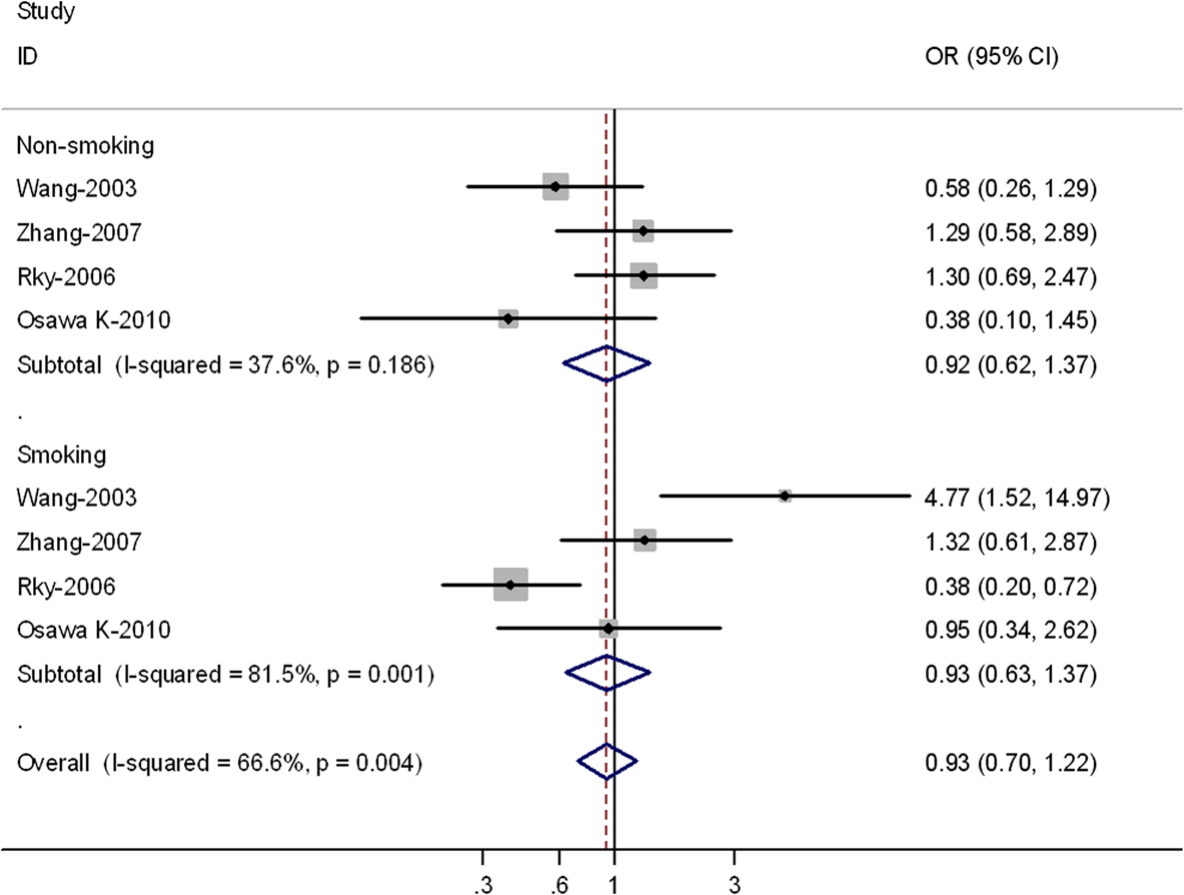
Forest plot (random-effects model) of lung cancer risk associated with XRCC3 Thr241Met polymorphisms for the (C/T + T/T) versus vs C/C stratified by smoking status of population.

In the subgroup analyses by smoking status, no significantly risks were found among smokers for (TC + TT) versus CC (OR = 0.93, 95% CI = 0.63-1.37; *P* = 0.001 for heterogeneity) or TT versus CC (OR = 0.98; 95% CI = 0.72-1.45; *P* = 0.006 for heterogeneity) (Table 4). In non-smokers, significantly risks were not found for (TC + TT) versus CC (OR = 0.92, 95% CI = 0.62-1.37; *P* = 0.186 for heterogeneity) or TT versus CC (OR = 0.99; 95% CI = 0.78-1.51; *P* = 0.230 for heterogeneity) (Figure 3).

**Table 4:** Distribution of XRCC3 Thr241Met genotypes among cases and controls stratified by smoking status

**First author-year**	**Ethnicity(country of origin)**	**Smoking status**	**Lung cancer cases**			**Controls**		
			**C/C**	**C/T**	**T/T**	**C/C**	**C/T**	**T/T**
Wang-2003(36)	USA (Mixed)	Non-smoking	24	10^#^		93	67^#^	
		Smoking	45	33^#^		26	4^#^	
Zhang-2007 (47)	China (Asian)	Non-smoking	73	12^#^		126	16^#^	
		Smoking	110	16^#^		118	13^#^	
Rky-2006 (35)	Sweden (Caucasian)	Non-smoking	31	53^#^		32	42^#^	
		Smoking	48	43^#^		24	56^#^	
Osawa K-2010	Japan (Asian)	Non-smoking	28	3^#^		42	12^#^	
		Smoking	63	9^#^		53	8^#^	

^#^, the number of the combined C/T and T/T genotypes.

### Sensitivity analyses

A single study involved in the meta-analysis was deleted each time to reflect the influence of the individual data set to the pooled ORs, and the corresponding pooled Ors were not materially altered (data not shown).

### Publication bias

**Figure 4:**
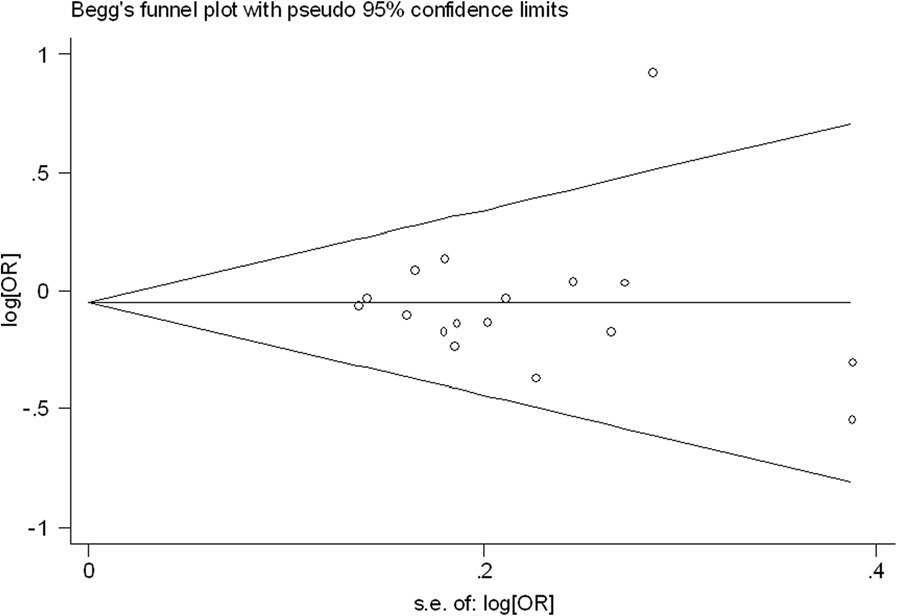
Begg’s funnel plot of XRCC3 Thr241Met polymorphisms for the (C/T + T/T) versus vs C/C for all studies.

**Figure 5:**
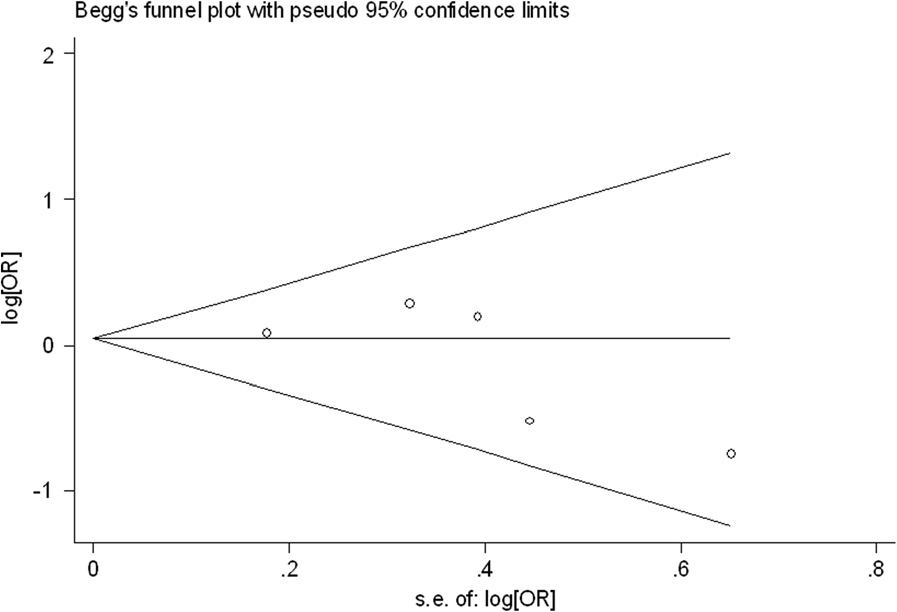
Begg’s funnel plot of XRCC3 Thr241Met polymorphisms for the (C/T + T/T) versus vs C/C stratified by histological types of lung cancer.

**Figure 6:**
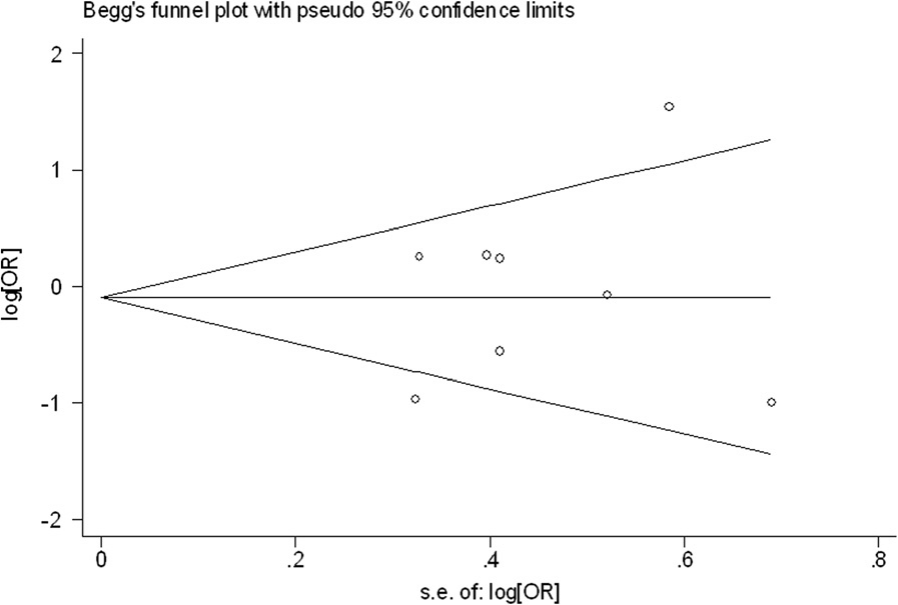
Begg’s funnel plot of XRCC3 Thr241Met polymorphisms for the (C/T + T/T) versus vs C/C stratified by smoking status of population.

Begg’s funnel plot and Egger’s test were performed to access the publication bias of literatures. Evaluation of publication bias for (TC + TT) versus CC for all studies showed that the Egger test was not significant (p = 0.927). For the subgroup analyses by histology, the Egger test was also not significant (p = 0.311) and for the subgroup analyses by smoking status, the p value of Egger test was 0.552. The funnel plots (Figures 4, 5, and 6) did not exhibit any patent asymmetry. These results indicated there was no evidence of publication bias in our meta-analysis.

## Discussion

It is well recognized that there is a range of individual susceptibility to the same kind of cancer even with identical environmental exposure. Host factors, including polymorphisms of genes involved in carcinogenesis may have accounted for this difference. Therefore, genetic susceptibility to cancer has been a research focus in scientific community. Recently, genetic variants of the DNA repair genes in the etiology of several cancers have drawn increasing attention. As it is known that individual studies with a small sample size may have not enough statistical power to detect a small risk factor, in this meta-analysis, we involved a total of 4123 lung cancer cases and 5597 controls and explored the association between the XRCC3 Thr241Met polymorphisms and lung cancer risk. Our results indicated that XRCC3 Thr241Met polymorphism was not significantly associated with the susceptibility to lung cancer. Additionally, no significant associations were also found in the stratified analysis by ethnicity, histological types or smoking status.

Population stratification is a troubling issue and can lead to spurious evidence on the association between markers and a disease, implicating the disparate effects of environment and ethnic differences on genetic background [32]. In this meta-analysis, ethnicity stratification of differences between Asians and Caucasians was not found. Tobacco smoke contains many known carcinogens and pro-carcinogens, such as benzopyrene and nitrosamine. Our meta-analysis results showed no significantly risks were found to be associated with the XRCC3 Thr241Met polymorphisms and lung cancer risk in smokers or non-smokers. There were only small number of studies examined the association between the XRCC3 Thr241Met gene polymorphism and lung cancer risk in smokers or non-smokers; moreover, the *p* value of Q test for heterogeneity test was significant. Considering the limited studies and P value of Q-test for heterogeneity test included in this meta-analysis, our results should be interpreted with caution.

When subgroup analyses by pathological type were considered, no significant associations were also found in lung AC subgroup or SCC subgroup. There are growing biological and epidemiological data to suggest that different lung cancer pathological subtypes, particularly the two most common, were distinct etiological entities that should be analyzed separately [33]. In the process of histological differentiation of lung cancer, XRCC3 Thr241Met polymorphisms may be not independent factor.

In our study, the three studies [17,19,25] accounted for 32.7% weight of all 17 studies. Popanda et al. [19] study accounted for 12.2% weight and included 921 cases, Lopez-Cima et al. [25] study accounted for 11.4% and included 837 cases, Misra et al. [17] study accounted for 9% and included 619 cases. The results of these three studies were consistent, with no significant association between the XRCC3Thr241 Met polymorphism and lung cancer risk. Moreover, the pooled OR of our meta-analysis was coincident with these three studies.

Improta G et al. [27] conducted a case–control study to examine the role of XRCC3 and XRCC1 genetic polymorphisms in the context of lung and colorectal cancer risk for Southern Italian population. As a result, the significant association was found between the XRCC3 Thr241Met polymorphisms and colorectal and lung cancer, more importantly, the risk of lung cancer of XRCC3 Thr241Met polymorphisms was relatively high (OR = 2.52, 95%: 1.44-4.41). In Wang et al. study [18], they found that no significant association between the XRCC3Thr241 Met polymorphism (OR = 1.04; 95% CI = 0.65–1.56) and lung cancer risk was shown. However, a significantly increased risk for lung cancer (OR = 4.77; 95% CI = 1.52 –14.97) was evident in smokers with the variant T-allele genotypes. Furthermore, a joint effect of the T-allele and heavy smoking was observed (OR = 37.31; 95% CI = 11.43–121.72). In our meta-analysis, for all studies the pooled OR was 0.95 (95% CI = 0.87-1.04), however the OR of the above-two studies was relative higher, thus they shown on the outlier of the Figures 1 and 3.

Some limitations of this meta-analysis should be acknowledged. First, heterogeneity can interfere with the interpretation of the results of a meta-analysis. Although we minimized this likelihood by performing a careful search of published studies, using explicit criteria for a study's inclusion and performing strict data extraction and analysis, significant interstudy heterogeneity nevertheless existed in nearly every comparison. The presence of heterogeneity can result from differences in the selection of controls, age distribution, and prevalence of lifestyle factors. Although most controls were selected from healthy populations, some studies had selected controls among friends or family members of lung cancer patients or patients with other diseases. Further, only published studies were included in this meta-analysis. The presence of publication bias indicates that non-significant or negative findings might be unpublished. Finally, our results were based on unadjusted estimates; a more precise analysis should have been conducted if individual data were available, which would have allowed us to adjust using other covariates, including age, ethnicity, family history, environmental factors, and lifestyle [34].

Despite these limitations, this meta-analysis suggests that the XRCC3 Thr241Met polymorphisms are not associated with lung cancer risk stratified analysis by ethnicity, histology and smoking status. However, it is necessary to conduct large sample studies using standardized unbiased genotyping methods and well-matched controls.

## Abbreviations

XRCC3: X-ray repair cross-complementing group 3; HRR: Homologous recombinational repair; OR: Odds ratio; NA: Not applicable.

## Competing interests

The authors declare no any conflicts of interest in this work.

## Authors' contributions

PZ and LKY contributed to the conception and design of the study, the analysis and interpretation of data, the revision of the article as well as final approval of the version to be submitted. QW and QQ participated in the design of the study, performed the statistical analysis, searched and selected the trials, drafted and revised the article. All authors read and approved the final version of the manuscript.
